# An mALBI-Child–Pugh-based nomogram for predicting post-hepatectomy liver failure grade B–C in patients with huge hepatocellular carcinoma: a multi-institutional study

**DOI:** 10.1186/s12957-022-02672-5

**Published:** 2022-06-16

**Authors:** Ming-Hao Xu, Bin Xu, Chen-Hao Zhou, Zhong Xue, Zhao-Shuo Chen, Wen-Xin Xu, Cheng Huang, Xiao-Dong Zhu, Jian Zhou, Jia Fan, Hui-Chuan Sun, Ying-Hao Shen

**Affiliations:** 1 Department of Liver Surgery and Transplantation, Liver Cancer Institute and Zhongshan Hospital, Fudan University, 180 Fenglin Road, Shanghai, 200032 China; 2 Department of Hepatobiliary Pancreatic Surgery, Fujian Medical University Cancer Hospital, Fuzhou, 350014 China

**Keywords:** Huge hepatocellular carcinoma, Modified albumin-bilirubin grade, Child–Pugh classification, Post-hepatectomy liver failure

## Abstract

**Objective:**

Post-hepatectomy liver failure (PHLF) is a severe complication in patients with hepatocellular carcinoma (HCC) who underwent hepatectomy. This study aims to develop a nomogram of PHLF grade B–C in patients with huge HCC (diameter ≥ 10 cm).

**Methods:**

We retrospectively collected clinical information of 514 and 97 patients who underwent hepatectomy for huge HCC at two medical centers between 2016 and 2021. Univariate and multivariate analysis were carried out to screen the independent risk factors of PHLF grade B–C, which were visualized as a nomogram.

**Results:**

Three Hundred Forty Three Thousand One Hundred Seventy One  and 97 HCC patients were included in the training cohort, internal validation cohort, and external validation cohort, with probabilities of PHLF grade B–C of 15.1%, 12.9%, and 22.7%, respectively. Pre-operative modified albumin-bilirubin (mALBI) grade (*p* < 0.001), Child–Pugh classification (*p* = 0.044), international normalized ratio (INR) (*p* = 0.005), cirrhosis (*p* = 0.019), and intraoperative blood loss (*p* = 0.004) were found to be independently associated with PHLF grade B–C in the training cohort. All the five independent factors were considered in the establishment of the nomogram model. In the internal validation cohort and external validation cohort, the area under receiver operating characteristic curve for the nomogram in PHLF grade B–C prediction reached 0.823 and 0.740, respectively. Divided into different risk groups according to the optimal cut-off value, patients in the high-risk group reported significantly higher frequency of PHLF grade B–C than those in the low-risk group, both in the training cohort and the validation cohort (*p* < 0.001).

**Conclusions:**

The proposed noninvasive nomogram based on mALBI-Child–Pugh and three other indicators achieved optimal prediction performance of PHLF grade B–C in patients with huge HCC.

**Supplementary Information:**

The online version contains supplementary material available at 10.1186/s12957-022-02672-5.

## Introduction

Primary liver cancer ranks the fourth leading cause of cancer-related death globally [1]. Hepatocellular carcinoma (HCC) accounts for more than 90% of liver cancer cases [2]. Symptoms of early-stage HCC are often insidious, thus the fact that some patients already develop huge HCC (diameter ≥ 10 cm) at the time of diagnosis [3]. Patients with huge HCC are considered to have poor prognosis because of the difficulties in R0 resection [4], which requires adequate margins and therefore demanding large extent of resection. Tumor shrinkage of neoadjuvant systemic therapy or preoperative locoregional treatment is one approach that may improve patient outcomes after hepatectomy for huge HCC [5, 6]. Association liver partition and portal vein ligation for staged hepatectomy (ALPPS) was also proven to be effective for treatment of huge hepatocellular carcinoma [7]. However, high prevalence of post-hepatectomy liver failure (PHLF) induced by the large resection extent and insufficient future liver remnant (FLR), in turn, limits the efficacy and safety of hepatectomy for huge HCC.

The concept of liver function reserve was initially established for maintenance of adequate postoperative liver function [8]. The International Study Group of Liver Surgery (ISGLS) developed the normative PHLF criteria in 2011 [9], and reducing the probability of PHLF has been among the purposes of preoperative evaluation thereafter.

To assess pre-hepatectomy liver function, serological test is one of the basic and noninvasive methods. Based on serological tests and clinical symptoms, Pugh et al. summarized the findings of Child and Turcotte and reported Child–Pugh classification in 1973 [10]. According to Child–Pugh classification, hepatectomy is considered relatively safe for patients in grade A, and somewhat helpful clinically. However, patients with Child–Pugh grade A may also differentiate greatly in liver function, with some still experiencing PHLF. Some studies also concentrate on the pathological severity of liver cirrhosis in patients with Child–Pugh grade A [11]. In comparison, albumin-bilirubin (ALBI) grade evaluation requires only two serological indices [12], while being precise, dynamic, validated in several studies in recent years [13, 14], and proven to be more effective in predicting prognosis in patients with compensated liver function than Child–Pugh classification [15]. As therapeutic modalities diversify in precision, Child–Pugh classification and ALBI grade are more frequently utilized for both surgical and nonsurgical treatment of liver cancer [16, 17]. So far, no evidence has shown the predictive value of Child–Pugh classification and ALBI grade for huge HCC patients with high PHLF probability. In the present study, we compared the significance of the Child–Pugh classification and ALBI grade in predicting PHLF grade B–C, and established a nomogram based on these two models in 343 patients with huge HCC who underwent radical surgery. The nomogram was further validated in independent internal and external cohorts.

## Methods

### Study population

Retrospective clinical information was collected for consecutive patients who underwent hepatectomy for HCC at Zhongshan Hospital, Fudan University and Fujian Medical University Cancer Hospital from January 2016 to December 2021. Patients enrolled in this study were screened based on the following inclusion and exclusion criteria. The inclusion criteria included (I) patients with huge HCC (maximum diameter of tumor extent ≥ 10 cm) confirmed by preoperative contrast enhanced magnetic resonance imaging (MRI) or computed tomography (CT) (Supplementary Fig. 1A and B); (II) radical resection with no tumor cells detected in the resection margins of the specimens under microscope and with re-examination of contrast enhanced MRI or CT around 1 month after hepatectomy, showing no evidence of residual disease [18] (Supplementary Fig. 1C and D); (III) complete records of preoperative and postoperative laboratory parameters and follow-up data. The exclusion criteria included: (I) previous history of liver surgery; (II) evidence of preoperative systematic or locoregional treatment; (III) evidence of macrovascular invasion or extrahepatic metastasis prior to surgery; or (IV) non-R0 resected HCC. In total, 514 eligible patients who underwent hepatectomy at Zhongshan Hospital, Fudan University (Xuhui District, Shanghai) between 2016 and 2021 were included in the study, and were divided into a training cohort and an internal validation cohort in the ratio of 2:1. Moreover, 97 eligible patients who underwent hepatectomy at Fujian Medical University Cancer Hospital (Fuzhou, Fujian Province) during the same period were included in the study as an external validation cohort.

The study was conducted in accordance with the ethical standards of the Helsinki Declaration and was approved by the Ethics Committees of Zhongshan hospital and Fujian Medical University Cancer Hospital. All patients have signed informed consent in written form before surgery.

### Definitions

HCC was diagnosed based on enhanced MRI or CT and validated by pathologic evidence, based on the guidelines for the Diagnosis and Treatment of Hepatocellular Carcinoma (2019 Edition) [18]. PHLF was evaluated as defined by the International Study Group of Liver Surgery (ISGLS) [9], which was diagnosed when the bilirubin level and international normalized ratio were simultaneously elevated 5 days after hepatectomy. Patients requiring no change in the clinical management are classified as PHLF grade A. In comparison, patients requiring noninvasive intervention including daily diuretics, albumin, and fresh frozen plasma, are classified as PHLF grade B, and those requiring invasive treatments including hemodialysis, extracorporeal liver support and liver transplantation, are classified as PHLF grade C. Therefore, PHLF grade B–C was considered as severe PHLF, and a key point of this study. Child–Pugh classification was evaluated according to the version developed by Pugh et al. summarizing the findings of Child and Turcotte and reported in 1973 [10]. ALBI scores were calculated as (0.66 × log10 bilirubin) + (− 0.085 × albumin) pre-operatively, where bilirubin was measured in μmol/L and albumin in g/L [12]. The grading brackets are specified as follows: ALBI score ≤  − 2.60 (mALBI grade 1), >  − 2.60 to ≤  − 2.27 (mALBI grade 2a), >  − 2.27 to ≤  − 1.39 (mALBI grade 2b), and >  − 1.39 (mALBI grade 3) [19, 20]. Hepatectomy was performed as previously described [21, 22]. Briefly, liver transection was performed with the clamp-crush technique or an ultrasonic dissector and used intermittent Pringle maneuver, i.e., 20 min of alternate block followed by 5 min of reperfusion. Ligation was carefully sutured at the site of hemorrhage or bile leakage. Hepatectomy was divided into major resection (more than three Couinaud segments) or minor resection (less than three Couinaud segments) resection. All preoperative variables were based on the most recent serologic test before surgery.

### Follow-up

Precisely, laboratory parameters were collected on postoperative days (PODs) 1, 3, 5, and 7, or more frequently as appropriate, including liver, kidney, and coagulation function tests. Medical records were also kept of the invasive or non-invasive treatments or operations during the postoperative hospital stay. PHLF was diagnosed by two experienced surgeons in the treatment team. Patients were routinely examined around 1 month after hepatectomy for AFP, DCP (PIVKA-II) and cancer antigen 19–9 (CA19-9) as well as by enhanced MRI or CT scans. After that, patients were requested to be examined every 3 months. Patients also received follow-up phone calls each year.

### Statistics

Statistics were analyzed using SPSS (v23.0; IBM, Armonk, NY, USA) and R software (v3.6.3; R Project for Statistical Computing). Categorical variables were demonstrated as counts and percentages, while continuous variables were summarized as medians (range) or means (standard deviation), as appropriate. Besides, Pearson’s *χ*^2^ analysis, Fisher’s exact test, or Mann–Whitney *U* test were adopted as appropriate, and maximum likelihood estimation (MLE) was adopted for logistic regression. To determine the predictors of PHLF grade B–C, multivariate regression analysis was performed using variables with *p* values of < 0.05 in univariate analysis, and a two-tailed *p* value of < 0.05 was considered statistically significant. The plotting of the nomogram, receiver operating characteristic (ROC) curves, calibration curves and decision curves were performed using the *rms* package, *pROC* package, and *rmda* package, respectively.

## Results

### Patient characteristics

The characteristics of patients in training cohort (*n* = 343), internal validation cohort (*n* = 171) and external validation cohort (*n* = 97) were shown in Table 1, with no differences found in baseline demographic variables, laboratory tests, and intraoperative major events between the training cohort and internal validation cohort. There were 16 (4.7%) Child–Pugh class B patients in the training cohort, and 5 (2.9%) in the internal validation cohort. Two hundred thirty-three (67.9%), 75 (21.9%), and 35 (10.2%) patients were classified as mALBI grade 1, 2a, and 2b in the training cohort, with 120 (70.2%), 41 (24.0%), and 10 (5.8%) patients classified as mALBI grade 1, 2a, and 2b in the internal validation cohort, respectively. The incidence of PHLF in the training cohort was 15.2%, which was comparable to that in the internal validation cohort (12.9%, *p* = 0.485).

**Table 1:** Preoperative information, intraoperative events and PHLF of patients in the training cohort (*n* = 343), internal validation cohort (*n* = 171), and external validation cohort (*n* = 97)

Variables		Training cohort (*n* = 343)	Internal validation cohort (*n* = 171)	External validation cohort (*n* = 97)
**Age, years**		56 (12.0)	54 (11.5)	51 (13.9)
**Sex**	Male	298 (86.9)	150 (87.7)	81 (83.5)
	Female	45 (13.1)	21 (12.3)	16 (16.5)
**Albumin, g/L**		40.6 (24.0–53.0)	38.0 (26.5–49.1)	41.2 (25.0–48.7)
**Total bilirubin, µmol/L**		12.4 (3.1–109.4)	13.6 (5.5–37.2)	12.0 (4.7–51.4)
**ALT, U/L**		32.4 (5.2–351.0)	31.0 (5.0–144.0)	32.4 (7.2–334.0)
**AST, U/L**		41.1 (13.0–251.4)	45.0 (14.0–599.0)	38.0 (10.4–308.0)
**γ-GT, U/L**		102.0 (16.0–1330.0)	94.0 (26.0–969.0)	93.0 (14.0–698.0)
**PT, s**		12.2 (9.4–19.0)	12.8 (10.9–18.0)	12.0 (10.1–15.2)
**INR**		1.08 (0.86–1.68)	1.04 (0.86–1.60)	1.07 (0.91–1.34)
**Hilar occlusion, min**		22 (0–79)	18 (0–96)	25 (0–90)
**Intraoperative blood loss, ml**		300 (20–4000)	650 (100–8850)	200 (20–2500)
**Extent of resection**	Major	308 (89.8)	158 (92.3)	87 (89.7)
	Minor	35 (10.2)	13 (7.6)	10 (10.3)
**Type of hepatectomy**	Laparoscopic	5 (1.5)	2 (1.1)	0 (0)
	Open	338 (98.5)	169 (98.8)	97 (100.0)
**Cirrhosis**	Yes	250 (72.9)	115 (67.3)	86 (88.7)
	No	93 (27.1)	56 (32.7)	11 (11.3)
**HBsAg**	Positive	241 (70.3)	127 (74.3)	75 (77.3)
	Negative	102 (29.7)	44 (25.7)	22 (22.7)
**HBeAg**	Positive	48 (14.0)	29 (17.0)	11 (11.3)
	Negative	295 (86.0)	142 (83.0)	86 (88.7)
**HBV DNA**	Positive	213 (62.1)	104 (60.8)	65 (67.0)
	Negative	130 (37.9)	67 (39.2)	32 (33.0)
**No. of tumors**	Single	191 (55.7)	103 (60.2)	66 (68.0)
	Multiple	152 (44.3)	68 (39.8)	31 (32.0)
**BCLC stage**	A	151 (44.0)	59 (34.5)	44 (45.4)
	B	74 (21.6)	28 (16.4)	19 (19.6)
	C	118 (34.4)	84 (49.1)	34 (35.1)
**AFP, ng/mL**	< 20	118 (34.4)	53 (31.0)	29 (29.9)
	20–400	76 (22.2)	41 (24.0)	17 (17.5)
	> 400	149 (43.3)	77 (45.0)	51 (52.6)
**mALBI grade**	1	233 (67.9)	120 (70.2)	36 (37.1)
	2a	75 (21.9)	41 (24.0)	40 (41.2)
	2b	35 (10.2)	10 (5.8)	21 (21.6)
**Child–Pugh scores**	A	327 (95.3)	166 (97.1)	91 (93.8)
	B	16 (4.7)	5 (2.9)	6 (6.2)
**PHLF, grade B–C**	Yes	52 (15.2)	22 (12.9)	23 (23.7)
	No	291 (84.8)	149 (87.1)	74 (76.3)

Categorical variables are summarized as *n* (%). Continuous variables are summarized as median (range) or mean (standard deviation), as appropriate. Pearson *χ*^2^, Fisher’s exact, and Mann–Whitney *U* (Wilcoxon rank sum test) tests were used, as appropriate

*AFP* α-fetoprotein, *mALBI* Modified albumin-bilirubin, *ALT* Alanine aminotransferase, *AST* Aspartate transaminase, *γ-GT* gamma-glutamyl transferase, *PT* Prothrombin time, *INR* International normalized ratio, *PHLF* Post-hepatectomy liver failure

### Establishment of the nomogram for PHLF grade B–C

**Fig. 1:**
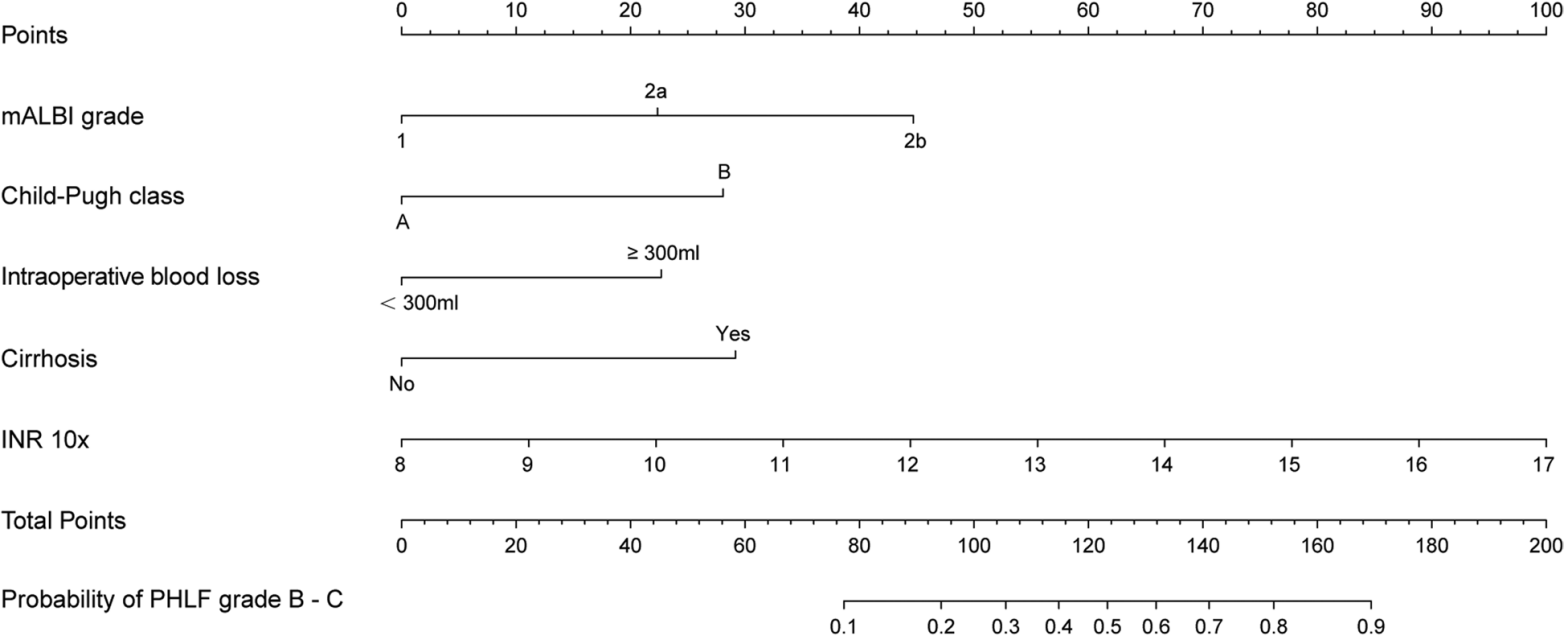
Nomogram for the prediction of PHLF grade B–C in patients with huge HCC developed in the training cohort. mALBI, modified albumin-bilirubin; INR, international normalized ratio; PHLF, post-hepatectomy liver failure

**Fig. 2:**
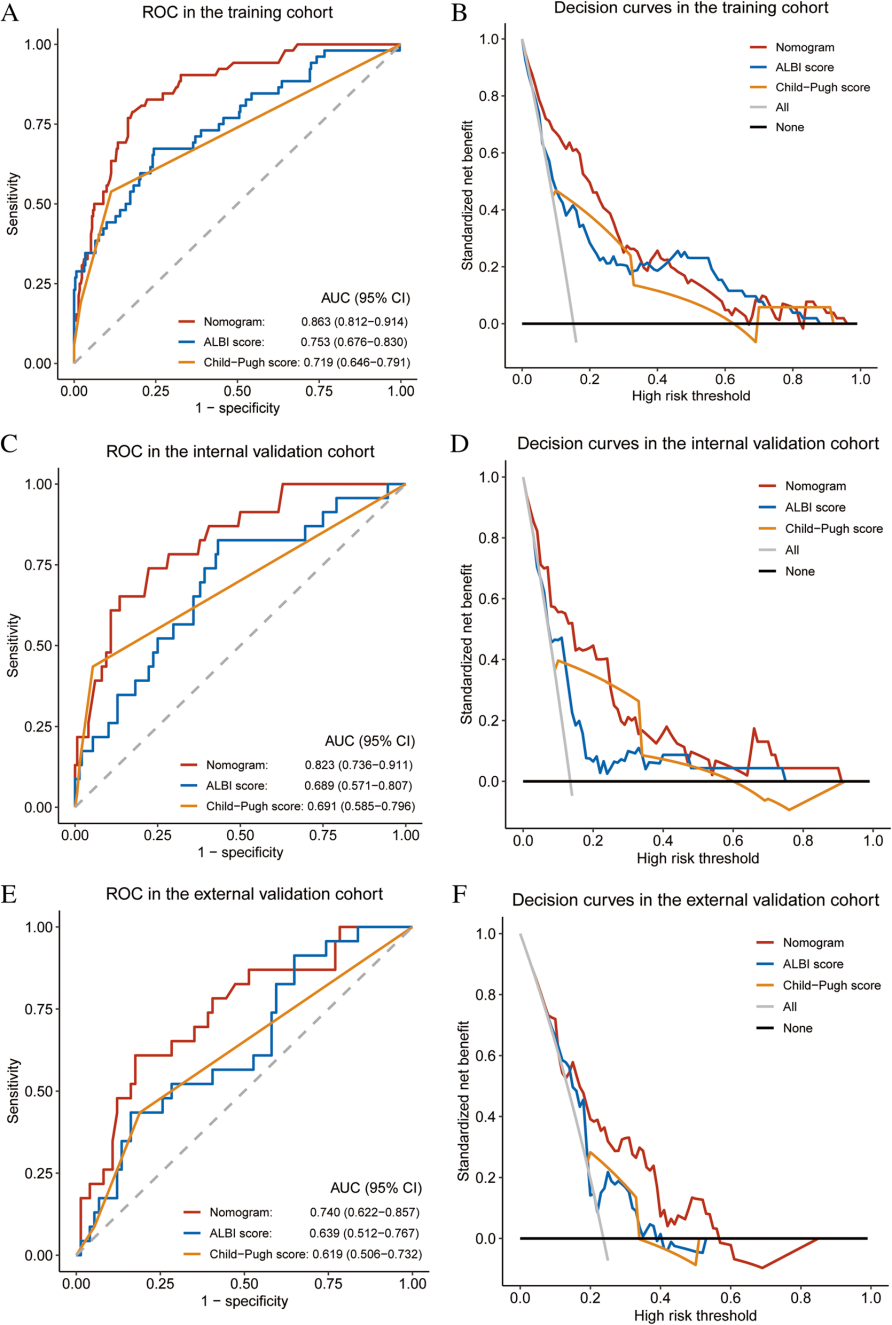
ROC curves for the nomogram in the training cohort (**A**), internal validation cohort (**C**) and external validation cohort (**E**), respectively. Decision curves for the nomogram in the training cohort (**B**), internal validation cohort (**D**) and external validation cohort (**F**), respectively. ROC, receiver operating characteristic; AUC, area under curve, ALBI, albumin-bilirubin

The results of univariate analysis were shown in Table 2. Precisely, the independent predictive significance for PHLF grade B–C was shown for international normalized ratio (INR), cirrhosis, intraoperative blood loss, Child–Pugh classification, and mALBI grade (Table 2). These independent risk factors were included in the establishment of the nomogram for severe PHLF prediction (Fig. 1) Receiver operating characteristic (ROC) analysis, decision curve analysis (DCA) and calibration curve analysis (CCA) were adopted to assess the predictive accuracy of the nomogram. The area under the ROC curve was 0.863 (*p* < 0.001, 95% CI, 0.812–0.914) for the nomogram, 0.753 (*p* < 0.001, 95% CI, 0.676–0.829) for ALBI scores and 0.718 (*p* < 0.001, 95% CI, 0.631–0.806) for Child–Pugh scores (Fig. 2A) Moreover, DCA suggested better predictive value of the nomogram than both ALBI score and Child–Pugh scores (Fig. 2B).

**Table 2:** Univariable and multivariable analyses for PHLF grade B–C in the training cohort

Variables		Univariable logistic regression				Multivariable logistic regression			
		β	*P* value	OR	95% CI	β	*P* value	OR	95% CI
**Age, years**		− 0.001	0.961	0.999	0.975–1.024		NA		
**Male gender**		− 0.035	0.937	0.966	0.406–2.296		NA		
**ALT, U/L**		0.006	0.055	1.006	1.000–1.011		NA		
**AST, U/L**		0.015	0.001	1.015	1.007–1.023		NS		
**γ-GT, U/L**		0.000	0.617	1.000	0.999–1.002		NS		
**PT, s**		0.661	0.001	1.936	1.461–2.565		NS		
**INR 10x**		0.914	0.001	2.493	1.733–3.506	0.529	0.005	1.698	1.198–2.501
**Hilar occlusion ≥ 20 min**		0.032	0.926	1.033	0.522–2.043		NA		
**Intraoperative blood loss ≥ 300 ml**		1.439	0.001	4.219	2.262–7.866	1.082	0.004	2.952	1.430–6.235
**Major resection**		1.909	0.063	6.747	0.903–50.413		NA		
**Open hepatectomy**		19.498	0.999	0.000	0.000–imponderable		NA		
**Cirrhosis**		1.665	0.002	5.287	1.850–15.109	1.392	0.019	4.022	1.397–15.037
**HBsAg positive**		0.279	0.418	1.322	0.673–2.597		NA		
**HBeAg positive**		1.024	0.005	2.785	1.370–5.662		NS		
**HBV DNA positive**		0.268	0.402	1.307	0.699–2.443		NA		
**Multiple tumors**		− 0.179	0.554	0.836	0.463–1.511		NA		
**AFP, ng/mL**	< 20		0.168				-		
	20–400	− 0.506	0.287	0.603	0.237–1.531		NA		
	> 400	0.318	0.343	1.375	0.712–2.655		NA		
**mALBI grade**	1		0.001				-		
	2a	1.315	0.001	3.724	1.791–7.744	1.087	0.009	2.966	1.297–6.765
	2b	2.599	0.001	13.453	5.887–30.745	2.126	0.001	8.383	3.197–22.508
**BCLC stage**	A		0.642				-		
	B	− 0.286	0.477	0.751	0.341–1.654		NA		
	C	− 0.282	0.413	0.754	0.384–1.482		NA		
**Child–Pugh B class**		2.426	0.001	11.310	3.908–32.732	1.343	0.044	3.830	1.057–14.844

*OR* Odds ratio, *CI* Confidence interval, *INR* International normalized ratio, *mALBI* Modified albumin-bilirubin, *AFP* α-fetoprotein, *ALT* Alanine aminotransferase, *AST* Aspartate transaminase, *γ-GT* gamma-glutamyl transferase, *PT* Prothrombin time, PHLF Post-hepatectomy liver failure, *NA* Not adopted, *NS* Not significant

### Validation of the nomogram in internal and external independent cohorts

**Fig. 3:**
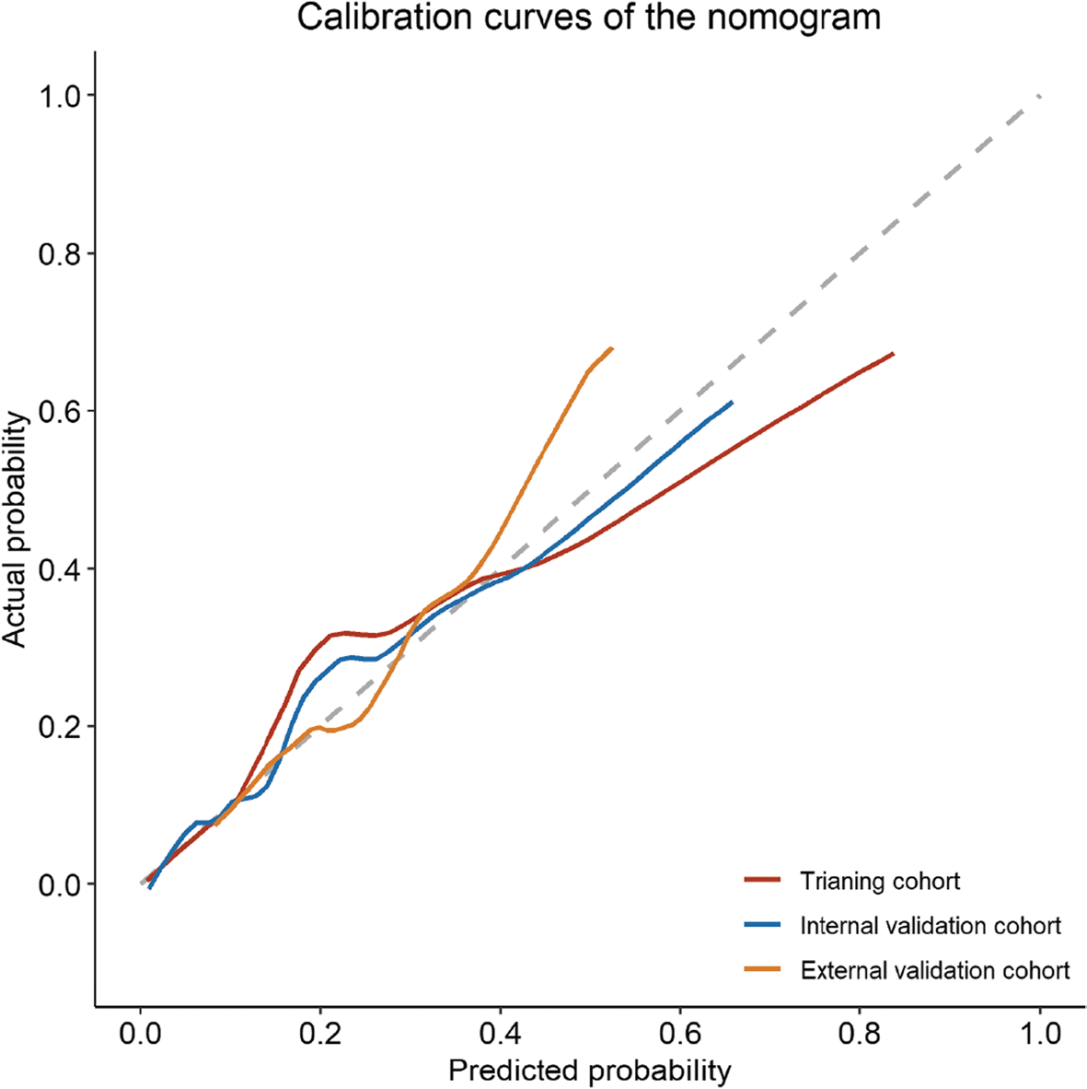
Calibration curves for the nomogram in the training cohort, internal validation cohort and external validation cohort, respectively

The area under the ROC curve was 0.823 (*p* < 0.001, 95% CI, 0.737–0.909), 0.689 (*p* = 0.004, 95% CI, 0.572–0.805), and 0.691 (*p* = 0.004, 95% CI, 0.555–0.827) for the nomogram, ALBI scores, and Child–Pugh scores in the internal validation cohort (Fig. 2C); and 0.740 (*p* = 0.001, 95% CI, 0.624–0.856), 0.639 (*p* = 0.044, 95% CI, 0.514–0.765, and 0.619 (*p* = 0.085, 95% CI, 0.482–0.857) for the nomogram, ALBI scores, and Child–Pugh scores in the external validation cohort (Fig. 2E). Consistent with the training cohort, both the internal and external validation cohorts demonstrated higher net benefit of the nomogram than the ALBI scores and Child–Pugh scores through the DCA curves (Fig. 2D, F). Calibration curve showed consistency between predictions and observations in the training cohort and the validation cohort (Fig. 3).

### Risk stratification of the nomogram

The nomogram score corresponding to the maximum Youden index was considered as the cut-off value in the training cohort. Patients with a nomogram score higher than 137.02 were considered as high-risk populations for PHLF grade B–C, and those with a lower nomogram score were considered as low-risk populations. Based on this, we pooled the probabilities of PHLF grade B–C for different risk groups in the training cohort and the validation cohort (both internal and external validation cohorts included) (Table 3). Patients in the high-risk group reported significantly higher frequency of PHLF grade B–C than those in the low-risk group, which was demonstrated both in the training cohort and the validation cohort (*p* < 0.001, *p* < 0.001; respectively).

**Table 3:** Incidence of PHLF grade B–C in high-risk group and low-risk groups in the training cohort and validation cohorts

Cohorts	Subgroups	PHLF grade B–C	*P* value
**Training cohort**	High-risk group	13 (68.4)	< 0.001
	Low-risk group	39 (12.0)	
**Validation cohort**^a^	High-risk group	8 (66.7)	< 0.001
	Low-risk group	38 (14.8)	

Data are presented as *n* (%). Fisher’s exact test was adopted

^a^Internal and external validation cohort were included

*PHLF* Post-hepatectomy liver failure

## Discussion

Hepatectomy remains the best treatment option for patients with HCC [23]. With advances in preoperative evaluation, intraoperative techniques, and postoperative management, the surgical indications for huge HCC have also expanded [24]. Especially, huge HCC may induce higher frequency of PHLF due to the large surgical resection extent. However, the studies on huge HCC were rather limited. Xiang et al. reported a probability of 29.0% of severe PHLF in 186 patients with huge HCC [25]. Guo et al. demonstrated that 13.8% patients experienced PHLF of all grades after major hepatectomy in a total of 745 patients [26]. In the present study, based on the experience of two centers over a period of 5 years, we reported a double PHLF grade B–C rate after hepatectomy in a large cohort of 611 patients with huge HCC.

PHLF grade B leads to deviation from regular clinical management and requires noninvasive treatment, while grade C requires invasive treatment [9]. Therefore, we sought for independent risk factors that could predict severe PHLF. Child–Pugh classification is widely acknowledged as a predictor for outcomes of liver surgery or transplantation [27–29]. However, the Child–Pugh classification was not precise enough, and assessment of ascites and hepatic encephalopathy was subjective. In recent years, there have been many studies looking further into predictors of hepatectomy in cohorts of Child–Pugh A patients [30, 31]. ALBI, together with other indicators, has been considered to improve the evaluation of liver function in recent years [32]. ALBI grade also showed good predictive ability for PHLF in HCC patients with different BCLC stages in previous report [33]. However, the ALBI grade also leaves much to be desired, as a large proportion of patients are classified as grade 2, while they differentiate significantly in actual liver function. A detailed assessment of ALBI grade, the mALBI grade, was therefore established by Kudo et al. [19]. Our results illustrated the similarly limited performance of Child–Pugh scores and ALBI scores in PHLF grade B–C prediction, which is possibly due to the influence of other preoperative and intraoperative characteristics. Among them, many characteristics may influence the development of HCC or prognosis of patients after hepatectomy [34, 35]. Therefore, we included some other baseline indicators and major intraoperative events in the univariate analysis, screening mALBI grade, Child–Pugh grade, intraoperative blood loss, cirrhosis, and INR as independent risk factors. A nomogram was established and validated in two independent cohorts for accurate prediction of PHLF grade B–C. Simple and noninvasive, the model has demonstrated its accuracy in PHLF grade B–C prediction in both the training and internal validation cohorts, and outperformed both Child–Pugh scores and mALBI scores separately. In an external validation cohort with nonidentical baseline patient characteristics, the model demonstrated adequate precision in predicting the occurrence of severe PHLF as well. Considering the results above, our nomogram model could potentially be generalized to other medical centers.

Also, the nomogram stratified candidates of hepatectomy for huge HCC into different risk groups of severe PHLF. In the overall cohort, 67.7% of the patients in the high-risk group developed severe PHLF, suggesting stricter standards on the surgical indications, as well as more careful perioperative management. For these patients, it might be safer to choose nonsurgical treatment [36], neoadjuvant systemic therapy [37], or TACE followed by hepatectomy [38]. In comparison with the high-risk group, patients in the low-risk group have a probability of 13.3% for developing PHLF grade B–C, thus considered more appropriate candidates for hepatectomy. Although the accuracy of this model still needs improvement, the stratification into different risk groups could benefit treatment choices for patients and surgeons.

The main limitation of this study is its retrospective nature with potential selection bias. Validation in more centers and larger cohorts is needed in the future. In addition, our cohort contained more than 70% patients with HBV infection, so whether the model can be applied equally to huge HCC due to other etiologies, including infection and steatohepatitis, should be explored in further studies.

## Conclusions

This noninvasive nomogram based on mALBI-Child–Pugh grade showed higher accuracy for predicting PHLF B–C compared with ALBI scores and Child–Pugh scores separately in patients with huge HCC, and could presumably improve indications for hepatectomy, and patient stratification for perioperative management by predicting PHLF grade B–C.

## Supplementary Information

**Additional file 1:** **Supplementary Figure 1.** (. (A, B)). Representative images of two huge HCCs by contrast-enhanced MRI. (C, D). Re-examination images around 1 month after hepatectomy of tumors in (A) and (B), respectively.

## Data Availability

The datasets during and/or analyzed during the current study are available from the corresponding author on reasonable request.

## Abbreviations

mALBI: Modified albumin-bilirubin
HCC: Hepatocellular carcinoma
PHLF: Post-hepatectomy liver failure
ALT: Alanine aminotransferase
AST: Aspartate transaminase
γ-GT: Gamma-glutamyl transferase
PT: Prothrombin time
INR: International normalized ratio
