# Supplementary figures and images for: An mALBI-Child–Pugh-based nomogram for predicting post-hepatectomy liver failure grade B–C in patients with huge hepatocellular carcinoma: a multi-institutional study
Source: World J Surg Oncol. 2022 Jun 16;20:206. doi: 10.1186/s12957-022-02672-5 (PMC9202189; doi:10.1186/s12957-022-02672-5)

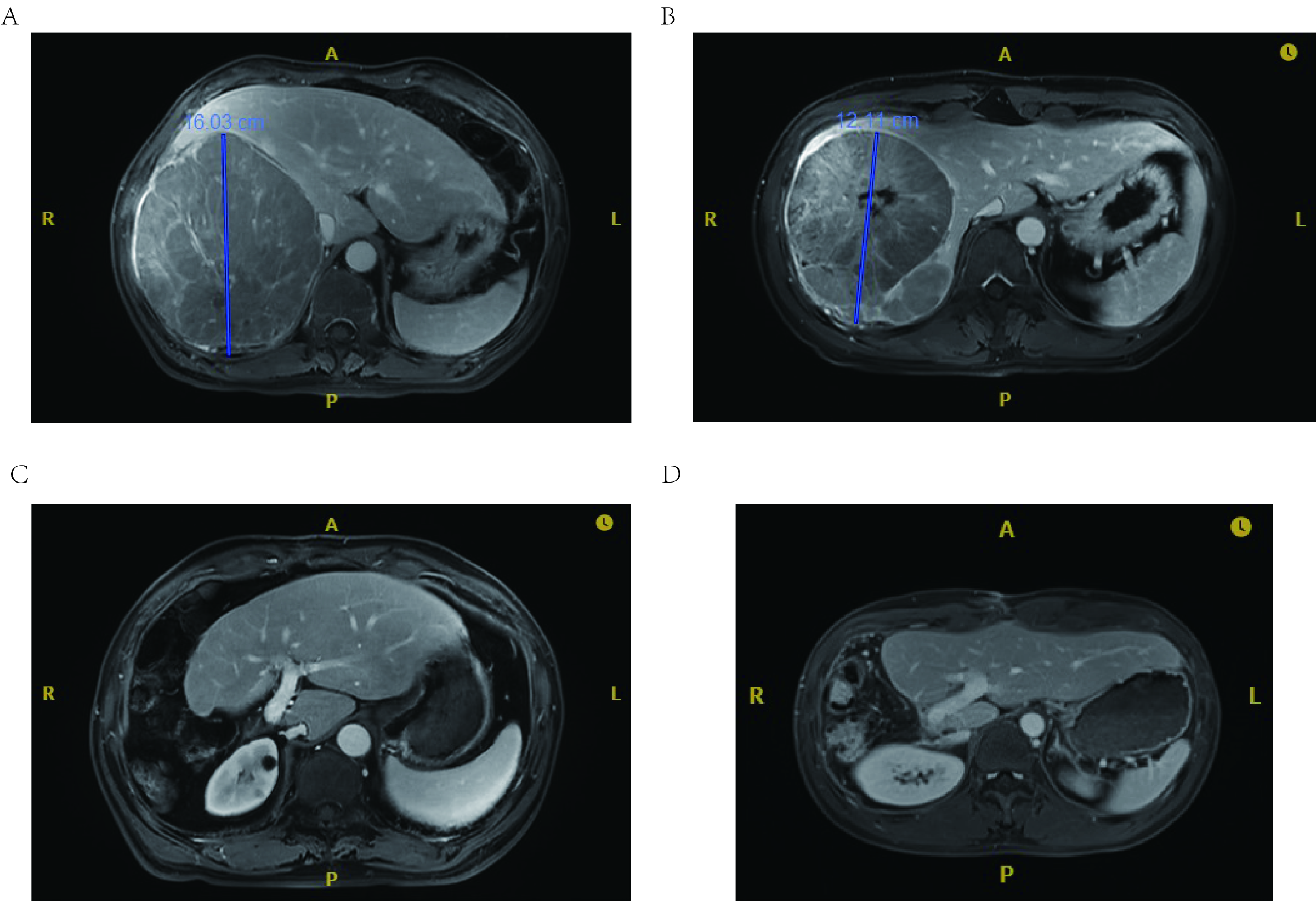

Supplement: Supplementary file 1 — Additional file 1: Supplementary Figure 1. (. (A, B)). Representative images of two huge HCCs by contrast-enhanced MRI. (C, D). Re-examination images around 1 month after hepatectomy of tumors in (A) and (B), respectively. [file 12957_2022_2672_MOESM1_ESM.tif]
